# Exploitation of *Trametes versicolor* for bioremediation of endocrine disrupting chemicals in bioreactors

**DOI:** 10.1371/journal.pone.0178758

**Published:** 2017-06-02

**Authors:** Cinzia Pezzella, Gemma Macellaro, Giovanni Sannia, Francesca Raganati, Giuseppe Olivieri, Antonio Marzocchella, Dietmar Schlosser, Alessandra Piscitelli

**Affiliations:** 1 Dipartimento di Scienze Chimiche, Università degli Studi di Napoli Federico II, Napoli, Italy; 2 Dipartimento di Ingegneria Chimica, dei Materiali e della Produzione Industriale, Università degli Studi di Napoli Federico II, Napoli, Italy; 3 Department of Environmental Microbiology, Helmholtz Centre for Environmental Research-UFZ, Leipzig, Germany; USDA Forest Service, UNITED STATES

## Abstract

Endocrine disrupting chemicals (EDCs) are environmental contaminants causing increasing concerns due to their toxicity, persistence and ubiquity. In the present study, degradative capabilities of *Trametes versicolor*, *Pleurotus ostreatus* and *Phanerochaete chrysosporium* to act on five EDCs, which represent different classes of chemicals (phenols, parabens and phthalate) and were first applied as single compounds, were assessed. *T*. *versicolor* was selected due to its efficiency against target EDCs and its potentialities were exploited against a mixture of EDCs in a cost-effective bioremediation process. A fed-batch approach as well as a starvation strategy were applied in order to reduce the need for input of ‘fresh’ biomass, and avoid the requirement for external nutrients. The fungus was successfully operated in two different bioreactors over one week. Semi-batch cultures were carried out by daily adding a mixture of EDCs to the bioreactors in a total of five consecutive degradation cycles. *T*. *versicolor* was able to efficiently remove all compounds during each cycle converting up to 21 mg L^-1^ day^-1^ of the tested EDCs. The maintained ability of *T*. *versicolor* to remove EDCs without any additional nutrients represents the main outcome of this study, which enables to forecast its application in a water treatment process.

## Introduction

In the last decades, a large number of materials and compounds have been produced and released into the environment without adequate knowledge on their interaction with the human health, and this behaviour resulted in a continuous pollution by a wide array of hazardous chemicals. EDCs are a group of compounds known to alter normal hormone regulation and to damage the health of living organisms and their progenies or subpopulations [1,2]. They are present in surface waters, groundwater, and even tap water due to their recalcitrance to activated sludge wastewater treatment [3]. Due to their widespread presence in the environment and toxic activity even at low concentrations, EDCs have received increased attention in water quality management and health care. Starting from the 2001, the European Commission launched chemical regulations to limit both their use and release in the environment. Since the beginning of 2000s, physical and chemical processes were used to remove such contaminants from wastewater, but they are commonly expensive and may generate a large volume of sludge [4]. On the other hand, biological wastewater treatments exploiting bacteria or fungi are attractive options as they could be cost-effective and environmentally friendly [5,6]. However, most of EDCs are present in the environment at a concentration level under the biodegradation threshold of bacteria [7] and /or are recalcitrant to their treatment [8]. In this context, ligninolytic white-rot fungi are increasingly being evaluated thanks to their ability to efficiently degrade a wide range of xenobiotics, such as dyes, chlorophenols and polycyclic aromatic hydrocarbons [9,10]. In most of the reported examples, this capability has been associated to the action of extra-cellular oxidative enzymes, well-known for the broad range of substrates they can attack [11]. Furthermore, some reports underline the better efficiency of fungal whole cell treatment compared to enzymatic ones [12], probably due to the synergic involvement of extracellular and mycelium-bound enzymes as well as biosorption phenomena [12]. In addition, these fungi may secrete low molecular weight redox mediators that enlarge the spectrum of oxidizable pollutants [13]. Nevertheless, the contribution of intracellular fungal enzyme systems has been also demonstrated for micropollutant degradation [14,15].

Experiments with purified ligninolytic enzymes have proven their ability to degrade EDCs [16–18]. Thus, the exploitation of fungi secreting ligninolytic enzymes into the extracellular medium represents a promising way for the biodegradation of these compounds [5,17,19–21]. A high number of studies have been conducted using different fungi, class of xenobiotics and culture conditions [5], however, it is difficult to make a comparison among data, thus specific relationships between these factors have not been analytically outlined. As a fact, the extent of removal depends on several factors including molecular structure of the contaminants, fungal species as well as their specific secreted oxidative enzymes and culture conditions [17]. It is worth to note that in all the studies a reduction of endocrine activity has been demonstrated [5, 21–24]. In most of the cases, the fungus *Trametes versicolor* has been proven as the most efficient to remove different classes of EDCs [17,21,25].

Most of the experiments on EDCs degradation by fungi have been performed in Erlenmeyer flasks, while only few studies have examined the degradation in bioreactors using *T*. *versicolor* fed with appropriate nutrients even in non-sterile conditions [26–30].

The current state-of-play emphasizes the need for further research, specially to properly design the right bioremediation strategy in terms of efficiency of the treatment and of process costs (*i*.*e*. bioreactor-operating; nutrient supply).

In this work, the abilities of three ligninolytic fungal strains such as *T*. *versicolor*, chosen according to its proven ability towards EDCs [17,21,25], *Pleurotus ostreatus* and *Phanaerochaete chrysosporium*, whose general biodegradative potential has been already verified [9], were exploited for the treatment of these micropollutants. Several typical representatives of EDCs were selected taking into account the respective amounts discharged per year [6,23]. Bisphenol A (BPA) is one of the most widely used chemicals in the world with production of 650,000 tonnes per year [31] and it is applied as an intermediate in the fabrication of polycarbonate plastics and epoxy resins [32]. Nonylphenol (NP) is present in polystyrene plastics and in the last 50 years it was extensively used as surfactant [33]. Parabens are esters of parahydroxybenzoic acid, widely used as preservatives in food, pharmaceutical, and cosmetic industries to prevent bacterial growth [34,35]. Two different parabens, *i*.*e*. methyl- and butyl-paraben (MTPRB and BTPRB) have been investigated in this study. Phthalates are a group of persistent, high production volume chemicals primarily used as additives in plastics, in order to make them more flexible [36]. Among various phthalates, this study focused on dimethyl phthalate (DMPTL).

The aims of the present study were to: *i)* compare the abilities of the three white-rot fungi *T*. *versicolor*, *P*. *chrysosporium* and *P*. *ostreatus* in contaminant removal using representative members of EDCs; *ii)* assess the potential of the best performing fungus (*T*. *versicolor*) towards a mixture of EDCs and under nutrient starvation; *iii)* test the reusability of *T*. *versicolor* biomass in repeated cycles of micropollutant additions; *iv)* demonstrate the exploitation of *T*. *versicolor* in the semi-continuous treatment of a EDCs mixture in bioreactor.

## Materials and methods

### Chemicals

EDCs were purchased from Sigma-Aldrich srl, Milano, Italy. BPA (MW = 228.29 g mol^-1^) was at 99% purity. NP used (MW = 220.35 g mol^-1^) was a technical mixture (tNP, 85% purity). MTPRB (MW = 152.15 g mol^-1^), BTPRB (MW = 194.23 g mol^-1^) and DMPTL (MW = 194.18 g mol^-1^) were HPLC grade. 1 mM stock of each EDC was prepared in hot deionised water. To improve the solubility of tNP and DMPTL, both methanol (0.4% v/v) and Tween 80 (0.1% w/v) were added. When an EDC mixture was used, a stock solution containing all compounds mentioned before was prepared and added to the culture broth.

#### Organisms and culture conditions

*T*. *versicolor* (NBRC4937) and *P*. *ostreatus* (Jacq.: Fr.) Kummer (type: Florida) (ATCC no. MYA-2306) were maintained through periodic transfer at 4°C on Potato Dextrose Yeast extract agar (24 g L^-1^ potato dextrose; 5 g L^-1^ yeast extract; 15 g L^-1^ agar) (PDY); *P*. *chrysosporium* Burdsall M1 (DSM 13583) was maintained through periodic transfer at 4°C on 10% PDY agar.

#### Fungal treatment of EDCs

In order to study EDCs removal, liquid fermentations using PDY medium were conducted. Preinocula for liquid cultures of both *T*. *versicolor* and *P*. *ostreatus* were prepared by inoculating 500 mL shaken flasks containing 300 mL of PDY with 6 agar plugs (11 mm diameter) containing mycelium grown on agar plates. Preinocula for liquid cultures of *P*. *chrysosporium* were prepared by inoculating 500 mL shaken flasks containing 300 mL of 10% PDY with 5 agar plugs (14 mm diameter) containing mycelium grown on solid state. After 5 days of growth in the dark at 28°C on a rotatory shaker at 150 rpm, preinoculum was homogenized and diluted 1:10 in 250 mL flasks containing 150 mL of culture broth supplemented with 150 μM copper sulphate and single EDCs (100 μM). Cultures were incubated in the dark at 28°C on a rotary shaker (150 rpm). Aliquots were daily withdrawn and cell-free samples assayed for oxidases and peroxidase activities and residual EDC concentration over 8 days.

#### Treatment of a mixture of EDCs by *T*. *versicolor* in rich medium (RM)

For treatment of a mixture of EDCs, *T*. *versicolor* preinoculum was homogenized and diluted 1:10 in 250 mL flasks containing 150 mL of PDY supplemented with 150 μM copper sulphate (rich medium, RM) and 20 μM of each EDC (thus corresponding to a final sum concentration of all EDCs of 100 μM). Cultures were incubated in the dark at 28°C on a rotary shaker (150 rpm). Aliquots were daily withdrawn and cell-free samples assayed for oxidases and peroxidase activities and residual EDC concentration over 8 days.

#### Treatment of a mixture of EDCs by *T*. *versicolor* in water medium (WM)

When experiments were performed in deionised water (water medium, WM), before homogenization, fungal mycelium from the preinoculum was filtered through cellulose nitrate filters (0.45 μm pore size; Sartorius), and then washed with sterile water, and then homogenized. Cultures were supplemented with 20 μM of each EDC. Aliquots were daily withdrawn and cell-free samples assayed for oxidases production and residual EDC concentration.

For experiments addressing the reuse of biomass in a fed batch approach, cultivation was conducted as above described. The EDC mixture was daily added at the defined concentration mentioned before. Cell-free samples were daily assayed for oxidases production and residual EDC concentration.

#### Control cultures

Cultures not supplemented with EDCs were always carried out as reference.

For biosorption quantification, flasks were inoculated with *T*. *versicolor* mycelium preinocula inactivated by autoclaving (at 121°C for 30 minutes), Tyndallisation (three 1 h cycles at 60°C with a 24 h interval between cycles), or by sodium azide (500 mg L^-1^) addition. Aliquots were daily withdrawn and cell-free samples assayed for residual EDC concentration.

### Tests in lab-scale bioreactors

**Fig 1:**
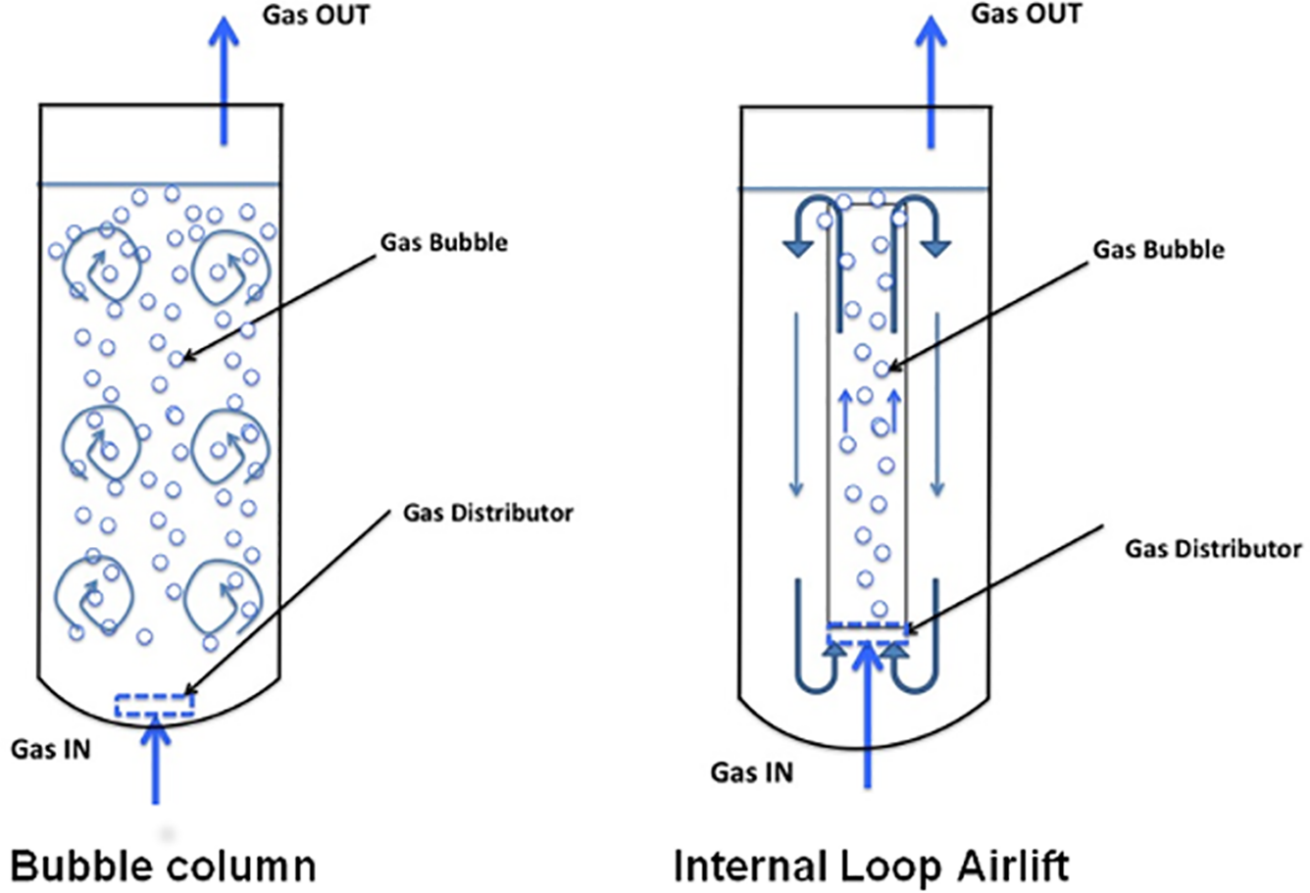
Sketch of the gas-liquid-solids bioreactor investigated. Liquid flow patterns are indicated.

Two lab-scale bioreactors were used: a bubbling column (BC) and an internal loop airlift (ILA) (Fig 1). The BC was a glass cylindrical column (40 mm ID, 800 mm high) equipped with an air distributor at the bottom. The working volume was set at 0.80 L. The ILA was a 40 mm ID, 200 mm high cylindrical column made of glass equipped with a coaxial 20/28 mm ID/OD, 150 mm high glass draft tube. Three glass branches held the draft along the centerline of the airlift and fixed the bottom clearance at 5 mm. The gas-liquid disengagement section was limited to the region just above the draft tube. The working volume was set at 0.30 L.

Reactors were sterilized at 121°C for 30 minutes. The fungal mycelium from the preinoculum was filtered and washed as described above, and used to inoculate WM supplemented with 20 μM of BPA, tNP, MTPRB and BTPRB (corresponding to a total concentration of EDCs in mixture of 80 μM). DMPTL was not used, considering its recalcitrance observed during recycling experiments in flasks. The bioreactors were operated under batch conditions with respect to the liquid and biomass phases, continuously with respect to the gas phase, and in fed batch mode with respect to the addition of EDCs. A mixture of EDCs (BPA, tNP, MTPRB and BTPRB; each at 20 μM) was applied: a small finite fraction of reactor liquid was daily replaced by an equal amount of fresh EDC solution in order to always achieve the desired initial concentration of EDCs (see above) without changing the final reactor volume. During operations sample ports enabled sterile sampling of the culture.

The bioreactors were operated at room temperature (25°C). The gas stream rate fed to the bioreactors was adequate for optimal biomass agitation/mixing, and minimization of foaming. Typically uniform loading of cells is achieved at very low gas stream rate [37].

Aliquots were daily withdrawn and cell-free samples assayed for residual EDC concentration.

### Assay of enzymatic activities

All enzymatic activities were monitored at 25°C. Laccase, Manganese peroxidase (MnP) and Lignin peroxidase (LiP) activities were assayed as previously described [38]. Aryl alcohol oxidase (AAO) activity was determined using veratryl alcohol as substrate. The reaction mixture contained 4 mM veratryl alcohol in 50 mM sodium phosphate buffer, pH 6.0. Oxidation of veratryl alcohol to veratrylaldehyde was followed by absorbance increase at 310 nm (ε_310_ = 9,300 M^-1^ cm^-1^).

### Analysis of residual EDC by High-Performance Liquid Chromatography (HPLC)

Cell-free samples were mixed with an equal volume of methanol and, after vigorous mixing, kept at -20°C for 15 minutes and centrifuged at 15,100 *g* at 4°C for 15 min. Then, 200 μL of obtained sample was analysed using a C18 column as previously described [18]. A concentration of 0.1 μM corresponds to the limit of detection of the EDCs in the applied conditions. The removed amount of each EDC was calculated considering respective purity.

## Results and discussion

### Comparison of white-rot fungi performances towards EDCs

**Fig 2:**
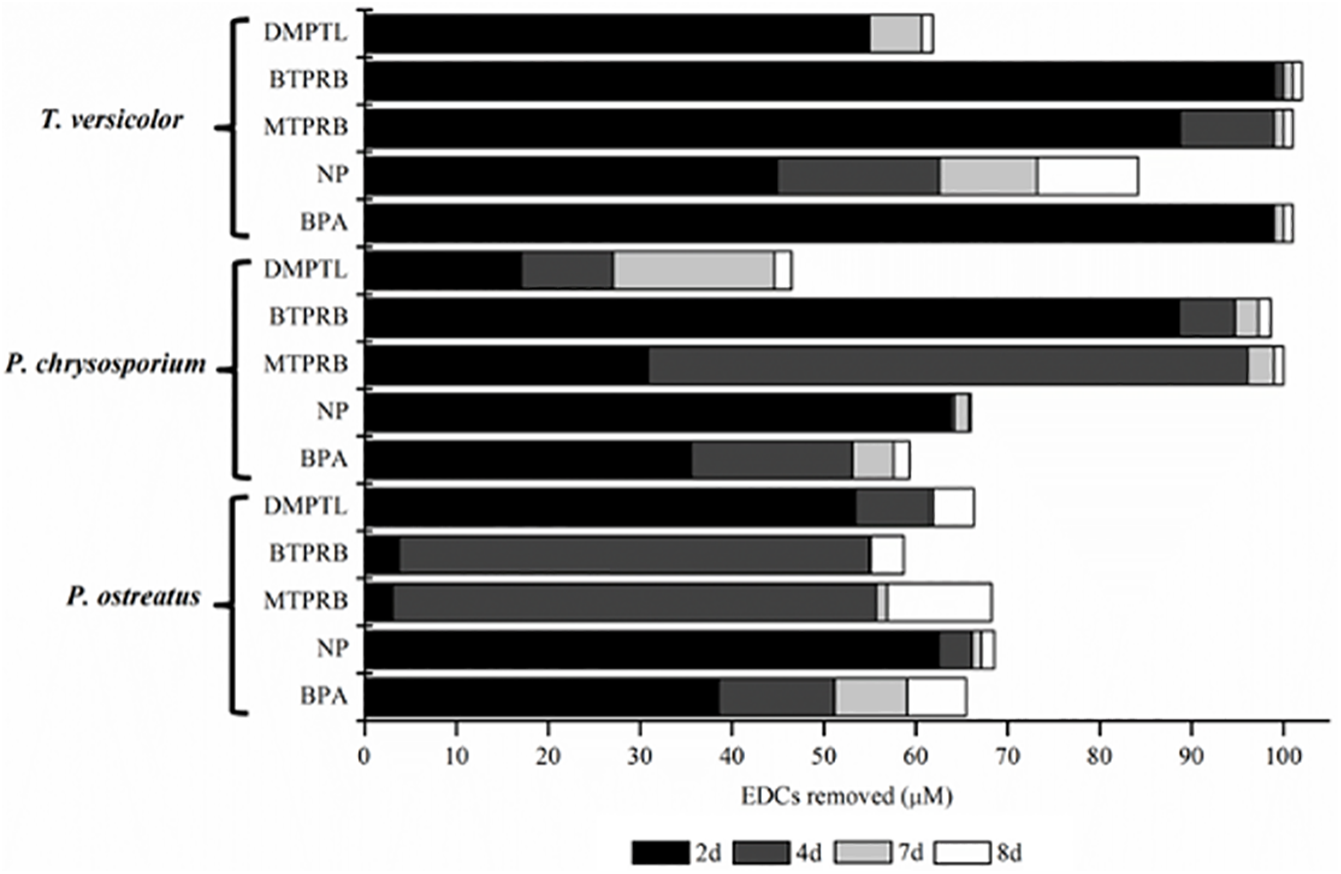
EDCs removed (μM) by *T*. *versicolor*, *P*. *chrysosporium* and *P*. *ostreatus* after 2, 4, 7 and 8 days of treatment in rich medium (RM). Single EDCs were applied to the culture broth at 100 μM, respectively. Standard deviations from three replicates of each series of results were less than ± 5%.

*T*. *versicolor*, *P*. *chrysosporium*, and *P*. *ostreatus* were tested for their degradative capabilities against selected EDCs. Fungi were able to grow in the culture broth supplemented with single EDC, displaying different rates of EDCs removal (Fig 2). *T*. *versicolor* gave the best results, being able to almost completely remove BPA and BTPRB after only 2 days, MTPRB after 4 days and tNP after 8 days. Conversely, DMPTL reduction level is about 60% after 8 days. *P*. *chrysosporium* almost completely removed BTPRB after only 2 days and MTPRB after 4 days, achieving a final removal comparable to that of *T*. *versicolor*. Whereas, tNP was removed up to 80% (68 μM, considering its purity) after 2 days, and no further disappearance was observed. A less efficient removal has been observed for BPA and DMPTL, reaching around 60% and 45% after 7 days, respectively. As far as *P*. *ostreatus* is concerned, 70% (60 μM) tNP disappearance was observed after 2 days, and no considerable improvement occurred. Its efficiency against all the other tested molecules was around 60% of removal. It is worth to note that tNP removal reached by *P*. *chrysospoprium* and *P*. *ostreatus* was initially faster than that obtained by *T*. *versicolor* (Fig 2), however only the latter fungus is able to completely remove this contaminant. As for DMPTL, *P*. *ostreatus* achieved the highest extent of removal, if compared to the other two fungi. Hwang and coworkers [20] ascribed this extent of removal to the production of hydrophobins. These low molecular weight proteins, also produced by the *P*. *ostreatus* [39] strain used in this study, could address hydrophobic phthalates on the mycelium surface, thus promoting their degradation by both intracellular and/or mycelium-associated enzymes [20]. Encouraging results were obtained for parabens with both *T*. *versicolor* and *P*. *chrysospoprium*, whereas until now only few examples of fungal treatment of these classes of molecules have been reported [40].

Peroxidases and AAO were assayed in the extracellular medium and barely detected. Concerning extracellular laccase activities, different activity levels were measured (S1 Fig). *P*. *ostreatus* secreted laccases in the presence of BPA, MTPRB and BTPRB, even if no xenobiotic-related induction was observed. A BPA inductive effect was instead recorded in the case of *P*. *chrysosporium*, since the fungus was able to secrete laccase enzymes only when grown in the presence of this molecule.

For *T*. *versicolor*, an inductive effect was detected for three out of the five EDCs applied (BPA, BTPRB, DMPTL) up to the fourth day of growth, with DMPTL causing the highest increase in laccase activity (7-fold increase at the second day of growth). Upon fungal treatment EDC concentrations may become under a threshold level triggering fungus response through the expression of enzymes [41].

Thus, even if no direct relationship between laccase secreted activities and EDCs removal could be inferred, these enzymes may contribute to the measured EDC removal in combination with both intracellular and mycelium-associated enzymes, as has already been reported before [14,15,20,42].

Thus, taking together the obtained results, *T*. *versicolor* is the most efficiently performing fungus in terms of the variety of the targeted EDCs, as well as of extent and rate of their removal.

### Potential of *T*. *versicolor* for EDC bioremediation

Considering *T*. *versicolor* performances, we aimed at fruitfully exploiting it in a cost-effective bioremediation process, using a mixture of xenobiotics, as well as a starvation strategy to avoid the requirement for external nutrients, and a biomass-fed-batch approach to reduce the need for input of ‘fresh’ biomass.

**Fig 3:**
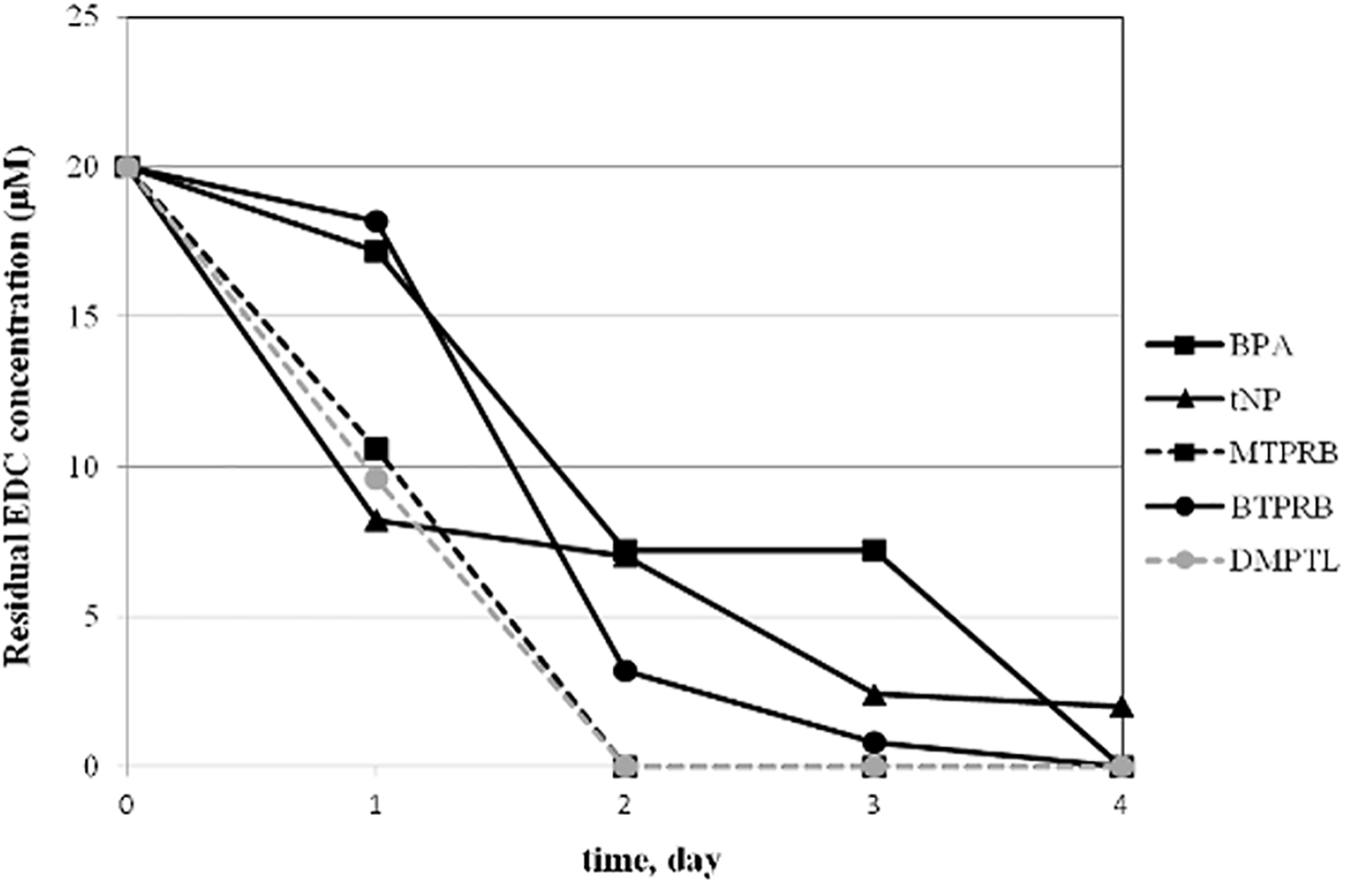
Residual EDCs concentration (μM) determined during treatment of EDC mixture by *T*. *versicolor* in RM. Standard deviations from three replicates of each series of results were less than ± 5%.

The ability of *T*. *versicolor* was evaluated by incubating the fungus in the presence of a mixture of EDCs (20 μM of each EDC, see sub-section on organisms and culture conditions). *T*. *versicolor* removed almost all the xenobiotics of the mixture within 4 days of treatment in RM (Fig 3). With the aim to evaluate the contribution of fungal biosorption, control experiments were carried out comparing the removal rates of active and inactive cultures. No detectable removal of EDCs was observed after inactivation by NaN_3_, Tyndallisation, or autoclaving. Thus it can be inferred that biotransformation is the main process responsible for their removal.

A further step ahead was performed assessing the fungal performance in water contaminated with the EDCs hereby re-using the same biomass in a total of sixteen total cycles of daily addition of EDCs. Surprisingly, when supplemented to WM, all xenobiotics of the mixture were completely removed after only one day, demonstrating an even faster removal rate than that obtained with rich medium. Four out of five molecules were completely removed after each cycle. On the other hand, DMPTL was completely treated only in the first cycle, whereas it accumulated during the following ones.

**Fig 4:**
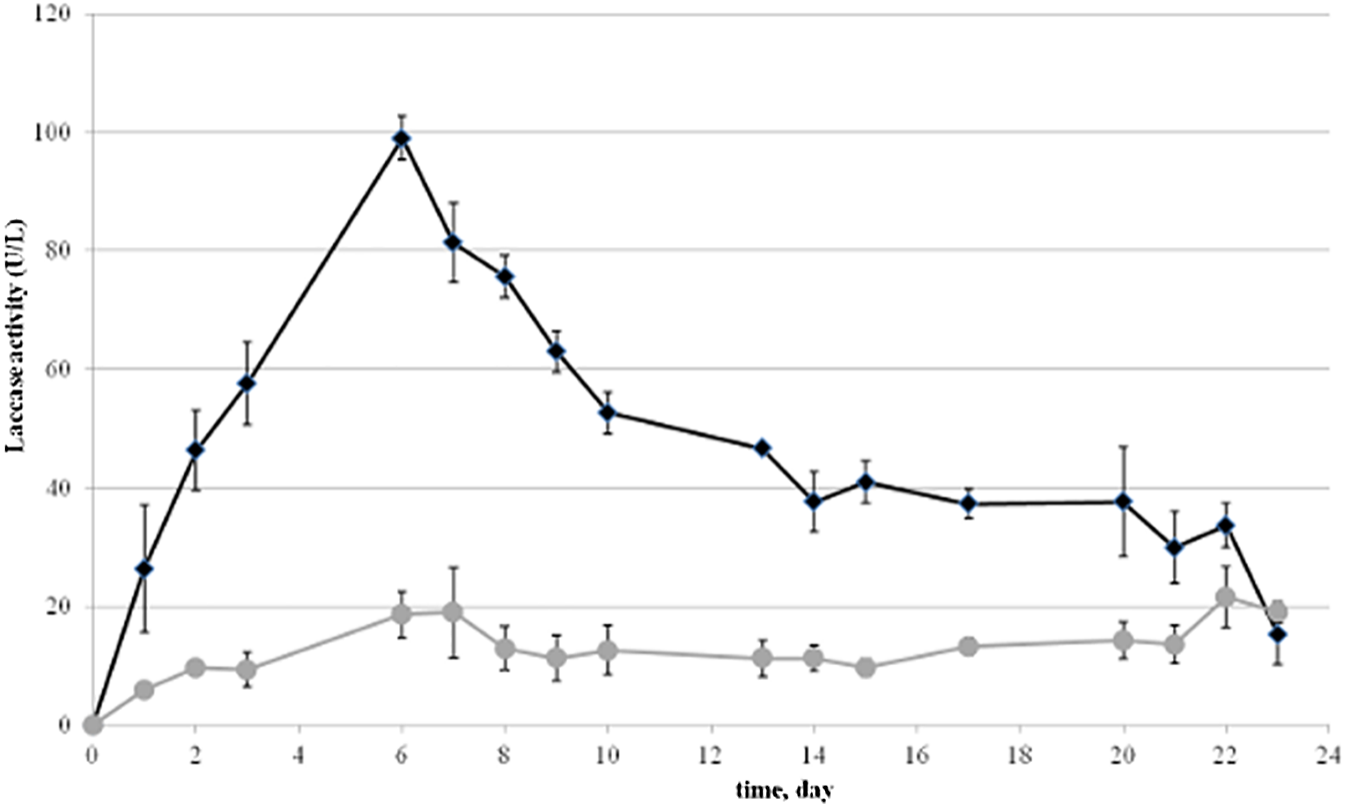
Laccase activity in the repeated cycles of EDCs degradation by *T*. *versicolor* in WM. Comparison of laccase activity in WM (grey circles) and EDCs supplemented WM (black diamonds).

Several oxidative enzyme activities were assayed in the extracellular medium and most of them were barely detected. A stimulating effect caused by EDCs was observed only for laccases. Increased extracellular laccase activity was observed in cultures supplemented with EDCs with respect to the control (Fig 4). Several reports documented the role of laccases and/or other oxidoreductases in degrading EDCs [16–18]. Proposed radical mechanisms involve the formation of phenoxy radicals from phenolic EDCs, which then undergo different non enzymatic reactions including oxidative coupling [23,43,44]. Evidence for both C-C and C-O coupling in the di- and oligomers derived from the action of laccases on NP and BPA has been reported [21–23].

The observed fungal performances may have benefited from the involvement of laccases, while the active contribution of mycelium-associated and intracellular enzymes also seems likely [14,15,20,42]. Indeed, the hydrophobicity of the analysed pollutants advocates for their cellular uptake *via* passive transport [21] The action of intracellular cytochrome P-450 systems in the fungal transformation of EDCs has been demonstrated [14,15,20,42]. Hydroxylated EDCs may enter further metabolism *via* β–oxidation [21]. A previous study has demonstrated that *T*. *versicolor* is able to use other emerging contaminants such as the UV blocker benzophenone-3 as a carbon source, whereas other emerging pollutants like the anti-inflammatory diclofenac do not contribute to fungal anabolism [3].

### Exploitation of *T*. *versicolor* for the removal of an EDC mixture in a semi-batch bioreactor

The promising results endorsed a further step towards implementation of the fungal system in lab-scale bioreactors. Two typologies of lab-scale bioreactors, namely a bubble column (BC) and an internal loop airlift bioreactor (ILA) were used (Fig 1). *T*. *versicolor* was incubated in WM containing four out of five EDCs (mixture of BPA, tNP, MTPRB and BTPRB). DMPTL was not used, considering its recalcitrance under the previous tested conditions. Semi-batch tests were carried out by daily adding the EDCs mixture to the bioreactors for four more times, thus corresponding to a total of five degradation cycles. The process is carried out in the absence of nutrients, in contrast with other studies describing the use of *T*. *versicolor* in bioreactor for the treatment of these classes of molecules in nutrient supplemented media [26–28].

**Fig 5:**
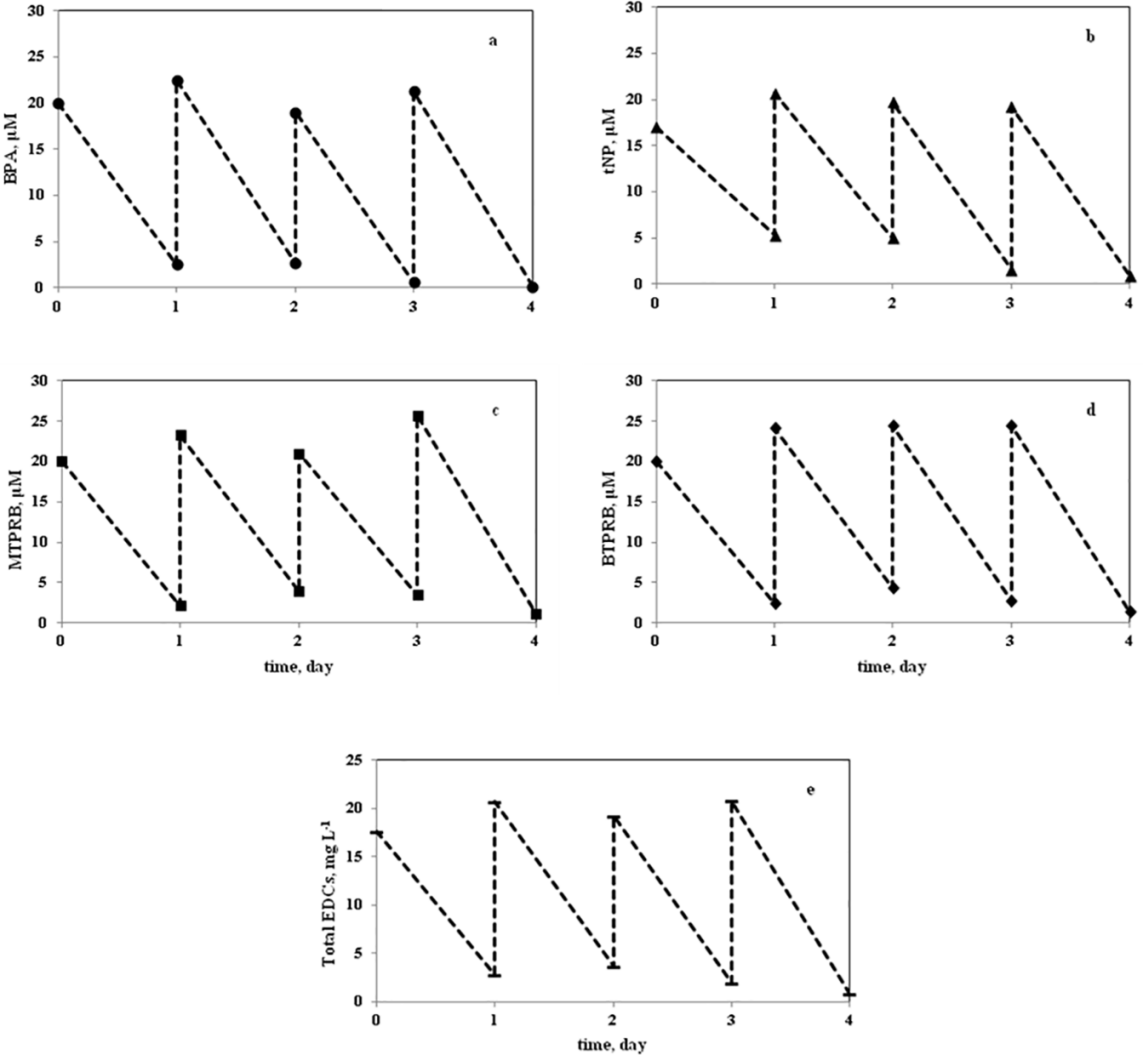
Concentration (μM) of individual EDCs (applied in mixture) (panel a-d) as measured in the bubble column bioreactor as function of the time. Panel e shows the sum concentration (mg L^-1^) of EDCs measured in the bioreactor. The concentration peaks correspond to the cyclic feeding with fresh EDCs.

The fungus was successfully operated in both bioreactors over a week. *T*. *versicolor* was able to efficiently remove all compounds during each cycle retaining almost the same efficiency. Fig 5 shows the concentration of each of the four EDCs measured in the mixture during a one week test in the 0.8 L BC bioreactor. The total concentration of the EDCs was also reported in the Fig 5 (Fig 5E). The conversion of each EDC was higher than 75% already at the end of the first cycle, and was practically complete starting from the third cycle. The concentration of each of the four EDCs measured in the mixture during one week tests in the 0.3 L ILA bioreactor confirmed those reported in the Fig 5. The analysis of the results of the two lab-scale bioreactors would notably suggest that neither the volume of the bioreactor or the shear stress—different for the two bioreactor typologies—affected the *T*. *versicolor* performances.

The obtained data pointed out that the bioreactors were successful to process up to about 21 mg L^-1^ day^-1^ of EDCs (Fig 5E) and in particular: 5.0 mg L^-1^ day^-1^ of BPA, 4.5 mg L^-1^ day^-1^ of NP, 6.0 mg L^-1^ day^-1^ of MTPRB, and 5.5 mg L^-1^ day^-1^ of BTPRB. It is expected that a larger EDC mass flow rate may be processed when the biomass concentration is increased.

## Conclusions

In conclusion, the obtained results show that all the fungi were able to remove the most of the tested contaminants displaying different efficiencies and preferences. The white rot fungus *T*. *versicolor* was selected due to its better behaviour against target EDCs. Fungal potentialities were exploited in bioremediation testing *T*. *versicolor* ability to adapt to more stressful conditions, such as the absence of nutrients and the concomitance of different xenobiotics. A further step towards utilization of this biosystem was pursued through reusing the fungal biomass for semi-continuous EDCs removal in bioreactor.

Our results demonstrated that *T*. *versicolor* efficiently removes EDCs without any needs of further nutrients over the investigated time period for repeated cycles. Biotransformation is the main process responsible for removal of EDCs, since negligible biosorption was detectable after NaN_3_-inactivation, Tyndallisation, or autoclaving, in contrast with other reports [45]. This may be due to the well-known differences among fungal surface hydrophobicity in response to varying growth conditions [45–48].

It remains to be elucidated whether the capability to remove EDCs in the absence of nutrients is related to cometabolism at the expense of fungal storage compounds, or whether some of the applied EDCs can be utilized as carbon and energy sources.

In this study the semi-continuous removal of EDCs without any nutrients supplementation was demonstrated re-using the same *T*. *versicolor* biomass for several cycles. Several factors makes this system attractive for application in real waste-water treatment:

- EDCs removal takes place in absence of nutrients giving a metabolic advantage to the fungus, letting to forecast its effectiveness also in non-sterile conditions;
- biomass recycling can be transferred to a continuous water treatment;
- the fungus does not require external nutrient supplementation, thus lowering the cost of the process;
- tests in bioreactors support the scalability of the process.

Taken together all these *pros* the application of this fungus in a continuous water treatment process after or in combination with conventional microbial treatment can be envisaged.

## Supporting information

S1 Fig **Laccase activity in *T*. *versicolor* (A), *P*. *chrysosporium* (B) and *P*. *ostreatus* (C) during single EDC (100** μ**M) treatment in rich medium (RM).** Control cultures were carried out in RM without EDC supplementation.
(DOCX)
