# Supplementary material for: Exploitation of Trametes versicolor for bioremediation of endocrine disrupting chemicals in bioreactors
Source: PLoS One. 2017 Jun 2;12(6):e0178758. doi: 10.1371/journal.pone.0178758 (PMC5456353; doi:10.1371/journal.pone.0178758)
Supplement: S1 Fig — Laccase activity in T. versicolor (A), P. chrysosporium (B) and P. ostreatus (C) during single EDC (100 μM) treatment in rich medium (RM). Control cultures were carried out in RM without EDC supplementation. (DOCX) [file pone.0178758.s001.docx]

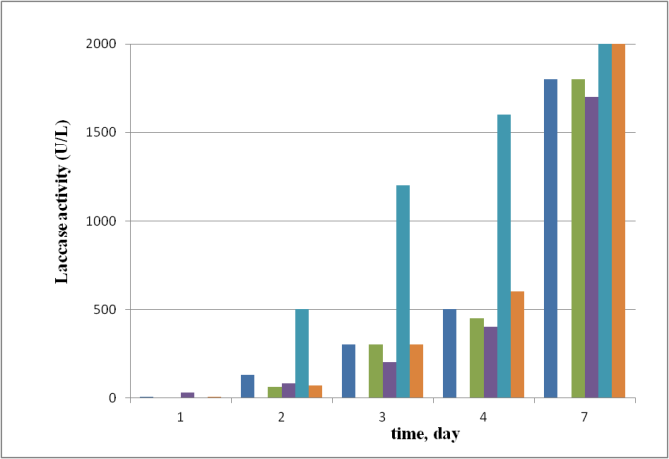

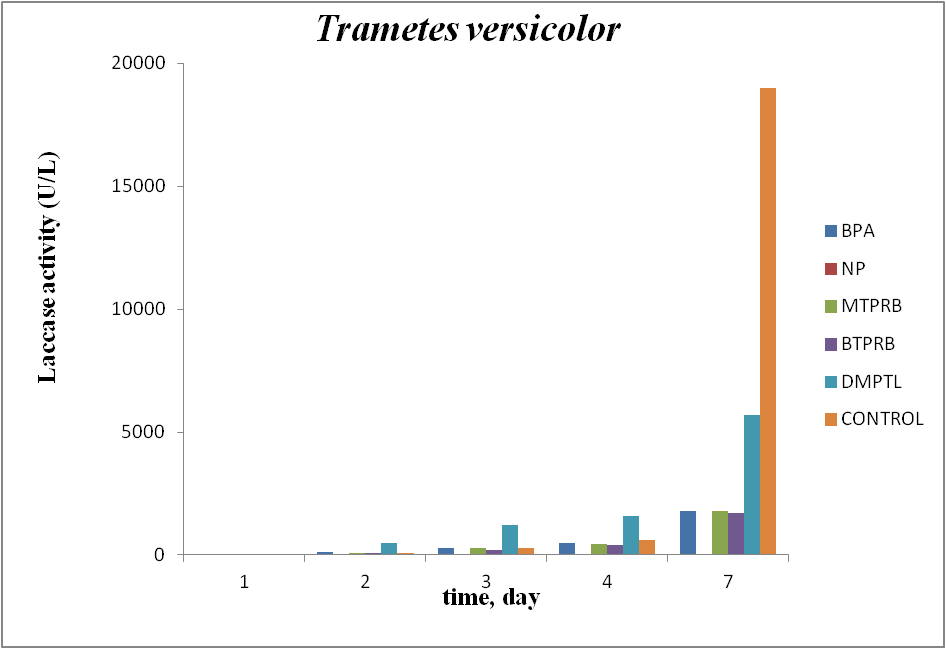


**A**


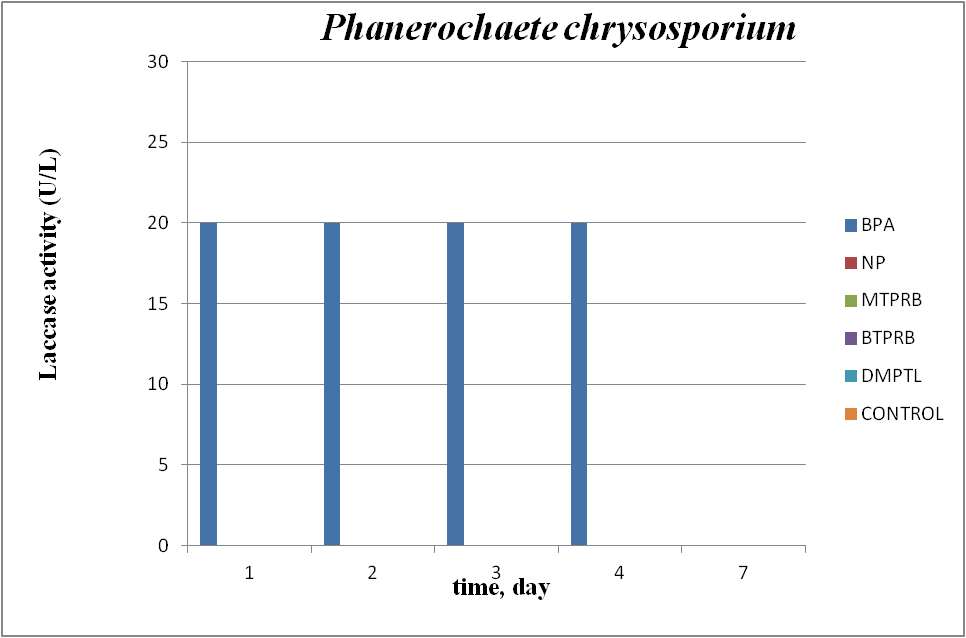


**B**


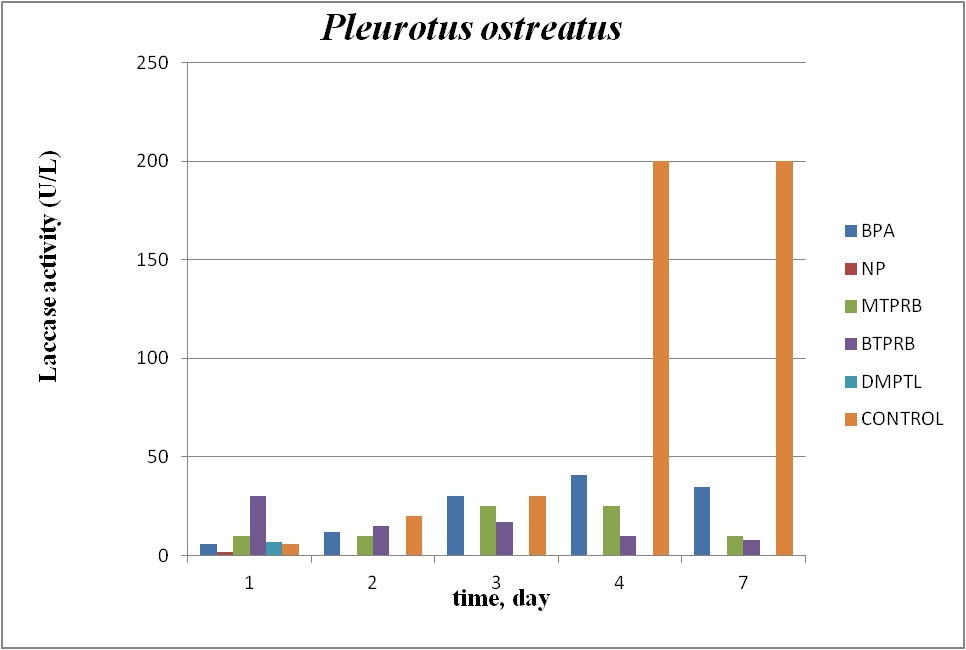


**C**

**S1 Fig. Laccase activity in *T. versicolor* (A), *P. chrysosporium* (B) and *P. ostreatus* (C) during single EDC (100 µM) treatment in rich medium (RM).** Control cultures were carried out in RM without EDC supplementation.
